# A Manual of the Modern Theory and Technique of Surgical Asepsis

**Published:** 1896-05

**Authors:** 


### Book Notices.

A Manual of the Modern Theory and Technique of Surgical Asepsis, by Carl Beck, M. D., Visiting Surgeon to St. Mark's Hospital and to the German Poliklinik of New York City, etc. 306 pages, with 65 illustrations in the text, and 12 full-page plates. Price, cloth, $1.25 net. W. B. Saunders, Publisher, 925 Walnut St., Philadelphia. 1895.

Dr. Beck, who is a teacher at the New York Post-graduate Medical School, has prepared this manual at the solicitation of those practitioners whom he has instructed at this institution and at St. Mark's hospital, and it is based upon the methods that he has employed in his teachings upon the treatment of wounds at these institutions.

The lack of full and detailed descriptions of the theory and technique of surgical asepsis, even in the best surgical text-books, makes a work of this kind necessary.

In detailing the management and treatment of wounds, the author draws a stricter line of demarcation than is usually done between wounds aseptically performed by the surgeon and those otherwise inflicted, or those dependent on inflammatory processes. In the latter class antisepsis is especially valuable, but only as subordinate to asepsis. The greater importance of asepsis is quite well stated in the author's words, as follows:

"While it should nearly always be possible to prevent infection of a wound made by a surgeon, it is practically impossible thoroughly to disinfect a wound in the common sense of the word, after infection has occurred. It is quite easy to sterilize an instrument or a towel in boiling water or in steam, but it is impossible to use such strong disinfecting agents on human tissue; nor can antiseptic solutions, even if they could be borne in full strength, directly permeate tissues which have been invaded by microbes."

The author covers the whole field of wound treatment, including influence of microbes, the importance of asepsis, means of disinfection, prophylactic disinfection, disinfection of instruments and dressings, sterilization of catgut, silk, etc.; sponges, drainage-tubes, and irrigation fluid, the aseptic operating room, aseptic wounds, infected wounds, aseptic open-wound treatment, renewal of dressings, technique of an aseptic operation, aseptic injection, anæsthesia, and asepsis in private practice.

He has described the best methods for staining and examining micro-organisms, the various apparatus, instruments, appliances, etc., necessary for this work, for operating and for use in aseptic dressings, etc. The book is profusely illustrated throughout. The publisher has evidently spared no expense to make the work the most useful and practical. H.

